# A multicentre parallel-group randomised trial assessing multiparametric MRI characterisation and image-guided biopsy of prostate in men suspected of having prostate cancer: MULTIPROS study protocol

**DOI:** 10.1186/s13063-019-3746-0

**Published:** 2019-11-21

**Authors:** Magdalena Szewczyk-Bieda, Cheng Wei, Katherine Coll, Stephen Gandy, Peter Donnan, Senthil Kumar Arcot Ragupathy, Paras Singh, Jennifer Wilson, Ghulam Nabi

**Affiliations:** 1 Department of Clinical Radiology, Ninewells Hospital, Dundee, DD1 9SY UK; 2 Division of Imaging Science and Technology, School of Medicine, University of Dundee, Ninewells Hospital, Dundee, DD1 9SY UK; 3 Tayside Clinical Trials Unit (TCTU), Tayside Medical Science Centre (TASC), University of Dundee, Ninewells Hospital, Dundee, DD1 9SY UK; 4 Department of Medical Physics, Ninewells Hospital, Dundee, DD1 9SY UK; 5 Division of Population Health Genomics, University of Dundee, Dundee, DD2 4BF UK; 6 Department of Clinical Radiology, Aberdeen Royal Infirmary, Aberdeen, AB25 2ZN UK; 7 Royal Free London NHS Foundation Trust, Royal Free Hospital, London, NW3 2QG UK; 8 Department of Clinical Pathology, Ninewells Hospital, Dundee, DD1 9SY UK

**Keywords:** Prostate cancer, Multiparametric magnetic resonance imaging, Prostate biopsy

## Abstract

**Background:**

There is growing evidence suggesting that multiparametric magnetic resonance imaging (mpMRI) is a marker for prostate cancer (PCa) aggressiveness and could be used to plan treatment. Improving early detection of clinically significant PCa with pre-biopsy mpMRI would very likely have advantages including optimising the diagnosis and treatment of diseases and diminishing patient anxiety.

**Methods and materials:**

This is a prospective multicentre study of pre-biopsy mpMRI diagnostic test accuracy with subgroup randomisation at a 1:1 ratio with respect to transrectal ultrasound (TRUS) and MRI/US fusion-guided biopsy or TRUS-only biopsy. It is designed as a single-gate study with a single set of inclusion criteria. The total duration of the recruitment phase was 48 months; however, this has now been extended to 66 months. A sample size of 600 participants is required.

**Discussion:**

The primary objective is to determine whether mpMRI can improve PCa detection and characterisation. The key secondary objective is to determine whether MRI/US fusion-guided biopsy can reduce the number of false-negative biopsies. Ethical approval was obtained from the East of Scotland Research Ethics Committee 1 (14/ES/1070) on 20 November 2014. The results of this study will be used for publication and presentation in national and international journals and at scientific conferences.

**Trial registration:**

ClinicalTrials.gov, NCT02745496. Retrospectively registered on 20 April 2016.

## Background

### Introduction and rationale

Men suspected of having prostate cancer (PCa) because of high levels of serum prostate-specific antigen (PSA) and/or abnormal digital rectal examination (DRE) are offered prostate biopsies guided by transrectal ultrasound (TRUS). Several challenges are seen in this crucial step. Firstly, the incidence of false-negative biopsies (i.e. clinically significant tumours not being detected) may be as high as 35% in first-time biopsies [1]; secondly, up to 44% of men younger than 70 years of age may need a second set of biopsies following initial negative results [2]; thirdly, up to 50% of PCa cases currently detected by these methods may not be clinically relevant when considering other patient-related factors; fourthly, data from UK Prostate Testing for Cancer and Treatment (ProtecT) has shown significant morbidity of the biopsy procedure, at times even leading to patient death [3]; and, finally, several studies have shown no improvement in PCa mortality, despite increased detection rates through screening programmes [4], and the number of deaths from PCa in the UK sadly remains largely unchanged.

Post-biopsy standard of care magnetic resonance imaging (MRI) is offered to patients with positive biopsy results or when there is a continuous increase in PSA levels despite negative biopsies. The performance of post-biopsy MRI in the staging and detection of PCa is significantly degraded by post-biopsy artefact [5, 6]. Therefore, it is recommended that MRI should be performed at least 10 weeks after biopsy, and if possible after 20 weeks [7]. Such a wait is unacceptable to patients and causes breaching of time with respect to treatment guidelines. Improving early detection of clinically significant PCa would very likely save many lives by refining the stratification of patients for optimising the treatment of significant disease, whilst simultaneously diminishing patient anxiety and morbidity through overdiagnosis and overtreatment.

### Objectives

The primary objective is to determine whether using multiparametric MRI (mpMRI) can improve cancer detection and the characterisation of PCa, and primary outcome measurements include:

- Number of PCa cases detected by mpMRI when compared to gold-standard prostatectomy specimens
- Number of clinically significant cancers detected by mpMRI when compared to gold-standard prostatectomy specimens

The secondary objective is to assess whether MRI/US fusion-guided biopsy can reduce the number of false-negative biopsies, and key outcome measurements include:

- Number of cancers detected in each randomised group, namely the intervention group (TRUS and MRI/US fusion-guided biopsy) vs. the standard of care group (TRUS only biopsy)
- Number of clinically significant cancers detected in each randomised group

### Trial design

**Fig. 1:**
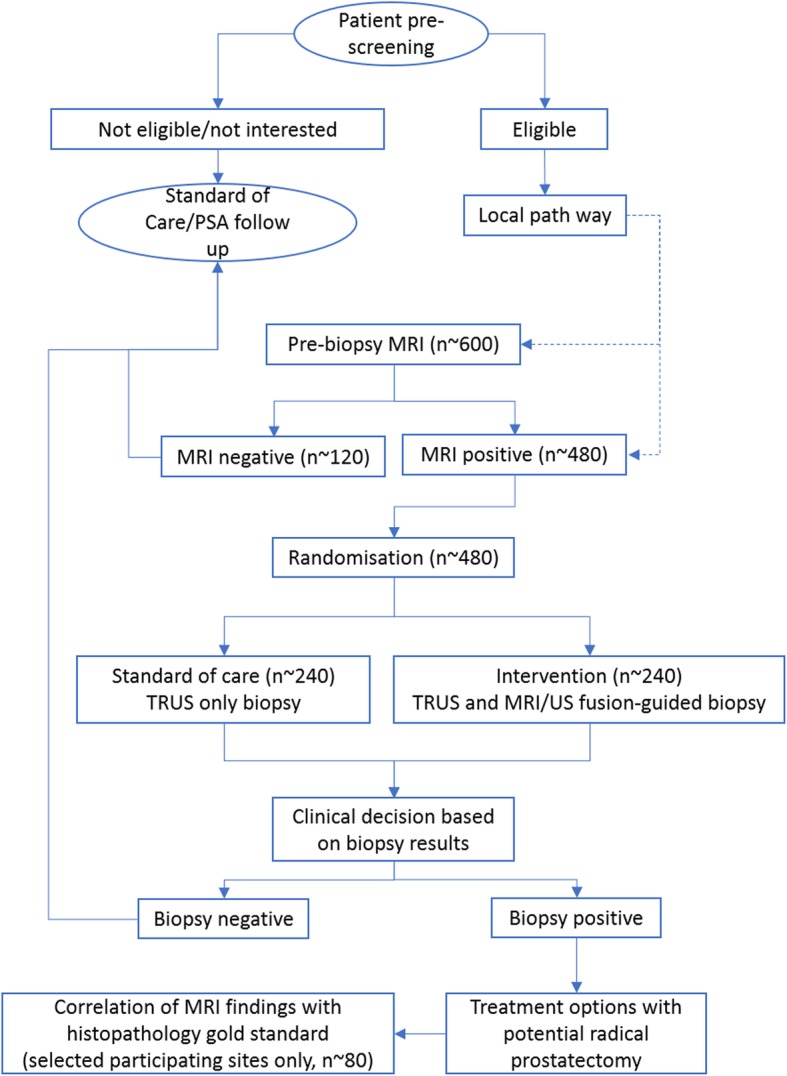
Design of the trial

This is a prospective multicentre study of pre-biopsy mpMRI diagnostic test accuracy with subgroup randomisation at a 1:1 ratio with respect to TRUS and MRI/US fusion-guided biopsy or TRUS-only biopsy. This is the first large trial to randomise men into either targeted or non-targeted biopsy with pre-biopsy MRI and to choose radical prostatectomy histopathology using 3D fabricated moulds as the gold standard (Fig. 1).

## Methods/design

### Study population and setting

Prospective recruitment of men referred with suspected clinically localised PCa will be carried out in urology clinics. The number of patients screened for eligibility over the recruitment period is estimated to be 1000. The number of patients not eligible for the study is estimated to be 350 and the number of patients who are likely to decline is estimated to be 50. The number of participants who are likely to progress to pre-biopsy MRI is estimated to be 600. Local audit information suggests that after a positive diagnosis of PCa, around 25% of men will opt for radical surgery. Based on the recruitment of 600 eligible men, the lowest amount of complete data for radical prostatectomy histology for analysis will be *n* = ~ 150. Our power calculation based on 80% sensitivity and a precision of ± 9% and an AUROC = 0.9 suggests that we need a minimum of 80 men with complete datasets from imaging and histopathology of radical prostatectomy to answer the primary objective. The dropout rate and incomplete datasets have been taken into consideration in designing this study.

Rais-Bahrami et al. [8] suggest that 80% of all pre-biopsy MRI scans (*n* = ~ 600) will have lesions suspicious for PCa, regardless of the level of suspicion assigned by mpMRI (*n* = ~ 480). Based on such information, we calculated that each of the two randomisation arms will have approximately 240 patients.

Initially, the total duration of the recruitment phase was 48 months; however, this will now be extended to 66 months with approval from the funding body. It is estimated that approximately 600 participants will be recruited and will receive a pre-biopsy MRI scan.

Participants will then be divided into two groups based on the mpMRI results. The group with positive mpMRI (*n* = ~ 480) will be randomised to the intervention (TRUS and MRI/US fusion-guided biopsy) group (*n* = ~ 240) or the standard care (TRUS-guided biopsy) group (*n* = ~ 240). The group with negative mpMRI (*n* = ~ 120) will undergo standard care (TRUS-guided biopsy) with histological comparative analysis. Participants will be followed through to a definite treatment stage. At sites where local facilities allow and for those who will undergo radical prostatectomy, histological findings will be compared to the pre-biopsy MRI results (*n* = ~ 80). The active trial participation for each participant will be varied depending on sites’ local pathways.

### Participant selection and enrolment

As standard care pathways differ among sites, the route of identification and screening of eligibility will differ in order to accommodate the local pathway. Potential participants will be identified by either the clinical team or the study team, with Caldicott Guardian Approval, depending on local practice.

#### Identifying potential participants

Potential participants may be identified through the following routes:

- At the urology clinic
- From GP referrals to the urology team
- By the urology team after standard care mpMRI (positive results only)

#### Contacting potential participants

Contact will be made by either the clinical team or the study team through the following routes:

- In person at clinics
  The clinical team will assess whether the patient meets the eligibility criteria for the study. If so, the study patient information sheet (PIS) will be given and a contact telephone number will be requested for a follow-up telephone call. A model PIS is shown in Additional file 1.
- Via letter
  The clinical urological team will review GP referral letters or positive mpMRI results and identify patients who meet trial eligibility criteria. Those who met the eligibility criteria will be sent a clinic appointment with the trial information leaflet.
- Via telephone call
  The clinical urological team or delegate may contact the patient and check whether he has received his NHS clinic appointment before sending out the information pack. After confirmation of receipt, the urological team or delegate (with appropriate Caldicott Guardian Approval) will introduce the study to the patient, and if they indicate interest, then an invitation pack will be sent.

If the patient declines the information pack or declines to provide a telephone number, no further attempt to recruit the patient will be made. The clinical or trial team will telephone patients who have agreed to be contacted. Any questions will be answered, and the patient will be asked whether he is interested in participating. The research team will arrange to see the patient for consent discussion. If required, the patient will be given additional time to consider participation before a final decision is made to arrange an appointment.

Patients will be advised to attend all of their planned scheduled appointments in the invitation letter and study introduction call. Prospective participants will be encouraged to telephone if they have any questions that they would like answered prior to attendance or to inform the research team if they change their minds with regard to participating. Patients who do not wish to participate will follow their local standard of care pathway.

#### Inclusion criteria

- Males aged 40–75 years at referral
- Had an MRI scan within 3 months prior to recruitment: for sites with pre-biopsy MRI included in the standard of care
- With at least 10 years’ life expectancy at clinical referral
- With clinically localised PCa: PSA ≤ 20 ng/ml
- And/or with abnormal DRE but < T3 disease
- Ability to give informed consent

#### Exclusion criteria

- Unable to give informed consent
- Prior prostatic biopsy within 12 months
- Contraindications to biopsy
- Poor general health and life expectancy < 10 years
- Previous diagnosis of acute prostatitis within 12 months
- History of PCa
- Prior transurethral prostatectomy
- Contraindications to MRI, including cardiac pacemakers, allergic reaction to gadolinium-based contrast, renal failure, intracranial clips, claustrophobia (applicable to participants who do not receive MRI as part of the standard care pathway)
- Previous hip replacement

#### Ineligible and non-recruited participants

Ineligible patients or those who decide not to enter the study will receive the standard of care. The reason(s) for ineligibility will be explained to all individuals who express interest in the study. Any questions they have will be answered. A record of the number of patients screened will be kept that indicates the number of patients eligible/ineligible to participate.

#### Blinding

In this study, blinding of the biopsy performer on men randomised to MR/US fusion-guided biopsy is not feasible, but radiologists and pathologists will be double-blinded regarding the patients’ outcomes until the end of the statistical analyses.

### Allocations and interventions

All enrolled participants will be offered 1.5 Tesla or 3.0 Tesla mpMRI prior to prostate biopsy where this is not already within the standard care pathway. A subgroup of participants with positive pre-biopsy MRI will be randomised into two further subgroups at a 1:1 ratio. Randomisation will be via TRUST, a web-based, good clinical practice (GCP)-compliant randomisation system run by the Health Informatics Centre (HIC). Randomisation will be implemented with random block sizes stratified by site and minimised by: the Prostate Imaging Reporting and Data System (PI-RADS) suspicion score (3–5); index lesion size (in two categories: above and below 6 mm in maximal diameter on MRI); age (40–59 years, 60–75 years); and PSA < 10.1 or PSA ≥ 10.1 to ≤ 20 ng/ml). The first subgroup will undergo a TRUS and MRI/US fusion-guided biopsy and the second will undergo standard of care TRUS-guide biopsies only. The MRI/US fusion-guided biopsy will be performed during the same attendance as the TRUS-guided biopsy***.*** TRUS and MRI/US fusion-guided biopsies will be performed by NHS-accredited radiologists and/or urologists from the research team. The persons undertaking the biopsy will have prior experience and will have received appropriate training from clinical experts and/or the equipment supplier’s application specialist(s). Those participants with negative pre-biopsy MRI will follow the local standard of care pathway; where this includes biopsy, data from the biopsy will be collected for analysis.

In a subgroup of patients who undergo radical prostatectomy, the prostatectomy specimen will be sectioned using customised moulds [9, 10] allowing for direct comparison with mpMRI. This will be carried out in NHS Tayside, NHS Grampian and other sites where local facilities allow. Comparison of mpMRI with a reference standard of radical prostatectomy pathology will be performed as the primary outcome.

### Withdrawal procedures

It will be made clear to patients that they can withdraw from the study at any time and return to the standard of care. However, any data collected up to the time of withdrawal will be included if participants agree to it.

### Proposed outcome and statistical analyses

#### Prediction performance (Objective 1)

The diagnostic accuracy of each of the three mpMRI predictive variables (T2WI (5-point ordinal scale), DWI (5-point ordinal scale) and DCE-MRI (2-point ordinal scale)) will be evaluated through logistic regression with PCa as the binary outcome (Yes/No). The T2WI and DWI will be dichotomised by assigning 0 to scores of 1–2 and assigning 1 to scores of 3–5 [11], and presented as percentages and denominators in the tables. The predictive performance of the dichotomised variables for the diagnosis of PCa will be analysed both individually and by considering all possible combinations among them. Additional variables as potential predictors in the logistic regression model will also include age, stage and comorbidity. Predictive ability will be estimated from the area under receiver operating characteristic (AUROC) curves drawn from the estimated probabilities given by the corresponding logistic regression models. Accuracy will be assessed by implementing the Hosmer–Lemeshow test for calibration. A score algorithm will be derived from the final logistic regression model.

#### Randomised controlled trial (Objective 2)

Analysis of the randomised comparison between MRI/US fusion-guided biopsy and standard TRUS-guided biopsy will be implemented according to the ICH E9 “Statistical Principles in Clinical Trials”. Analysis will be based on the intention-to-treat principle. The key outcome for the randomised study is to measure whether the MRI/US fusion-guided biopsy can improve the detection of cancer foci and to establish the reliability of the pre-biopsy MRI for the localisation of cancer foci within the prostate gland.

Secondary outcomes will include the following:

- PCa detected on fusion biopsy which is missed on TRUS biopsy
- Safety outcomes of death, and side effects such as pain and bleeding with the severity and duration of symptoms recorded in each randomised group
- Comparison of MRI-negative standard of care TRUS-guided biopsies with MRI-positive TRUS histopathology to facilitate analysis of the diagnostic accuracy of MRI in men suspected of a target condition

Binary outcomes such as cancer (Yes/No) and pain (Yes/No) will be analysed using logistic regression with the intervention arm as a binary variable in the model. All analyses will be stratified by site and minimisation variables, and adjusted for prostatic volume and maximum lesion size. Outcomes such as the Gleason Score (GS) and the number of lesions (depending on the range of values obtained) will be analysed as continuous linear regression or ordinal logistic regression. Subgroup analyses will be carried out as secondary analyses by adding treatment by subgroup variable interaction terms in the regression model. Analyses will be conducted by the study statisticians utilising SAS (9.4).

### Data collection and management

The case report form (CRF) will be developed together with the trial management team, statistician and data manager to ensure that the data management system supports the research aims of the study. The data management system will be fully validated, including the provision of test data and supporting documentation. It is the CI’s responsibility to ensure the accuracy of all data entered and recorded in the CRF/Electronic Data Capture System (EDCS). Data will be stored on servers controlled by the HIC and housed at the University of Dundee. Back-up and disaster recovery will be provided by the HIC according to its standard operating procedures. The data in the CRF will be stored by the MULTIPROS study group for 5 years after the end of study. Future information on the EDCS is available upon request to the HIC.

### Trial management, monitoring and auditing

#### Study management

Tayside Clinical Trial Unit, a UKCRC-registered trials unit, will be responsible for overseeing management of the study. A dedicated trial-specific manager will run the day-to-day work of the study and report to the CI. Specifically, the trial manager, in consultation and close working with the CI, will ensure the completeness and consistency of CRFs. The study management team will discuss any query and resolve it with input from the CI. The CI may choose to delegate some of the tasks to a member. A detailed trial-specific Delegation Log will be made available at each recruiting site, with clear mention of the tasks each member of staff will be taking part in. The trial management group (TMG) consists of the Chief and co-investigators and representatives from each participating site. The TMG meets monthly to keep track of progress with recruitment and resolve challenges as and when they arise.

#### Joint Data Monitoring Committee and Trial Steering Group Charter

A Trial Steering Committee (TSC) with an integrated Data Monitoring Committee (DMC) will be put in place. The combined group will perform a patient safety review and provide overall strategic direction and supervision during the progress of the study. The DMC is integrated because the foreseen risk profile of the study is minimal. Membership will consist of a lay-member of the patients group as Chair and a minimum of two other experts in the area of trial management or treatment of PCa. The CI will participate as invited by the TSC/DMC. The independent committee members will ensure research governance, review data and suggest alterations to the study based on risk–benefit analyses of the data. The later may include early termination of recruitment and other modifications as desired. The terms of reference will be agreed upon to clarify the membership, roles and responsibilities of the joint committee. All of the processes and procedures will be documented in the MULTIPROS Joint Committee Charter.

#### Inspection of records

The CI, PIs and all of the participating sites for recruitment in the study will undertake a commitment to allow free study-specific monitoring, periodic audits and a Research Ethics Committee (REC) review. It is expected that the CI will sign an agreement to allow direct access to all documentation and study-related records and material for the study Sponsor or a representative of the Sponsor whenever needed.

#### End of study

The end of study for a participant is the date at which the biopsy procedure was performed plus 30 days. The definition of the end of the study date for this trial is database locked. The Sponsor, CI and/or management group reserve the right to close the study to recruitment for any reasons including safety of the participants, less than satisfactory infrastructure or lack of progress. If the decision is taken to terminate the study prematurely, the decision on the end of the study will be communicated to the Sponsor and REC within 90 days, or 15 days of the decision. The CI will be responsible for any further follow-up of the participants and their safety. The Sponsor and REC will be informed and a summary report of the study will be submitted to both within 1 year of the decision to end the study.

### Harms

As the study does not employ an investigational medicinal product, adverse events (AEs) and serious adverse events (SAEs) will be recorded and reported as per the Health Research Authority (HRA).

An SAE is defined as an untoward occurrence that:

- Results in death
- Is life-threatening
- Requires hospitalisation or prolongation of existing hospitalisation
- Results in persistent or significant disability or incapacity
- Consists of a congenital anomaly or birth defect
- Is otherwise considered medically significant by the investigator

An SAE occurring for a research participant will be reported to the main REC and Sponsor where, in the opinion of the CI, the event is: related, it results from administration of any of the research procedures; and unexpected, the type of event is not listed in the protocol as an expected occurrence.

Due to the clinical status of the study population we will record in the CRF, but not report to the REC or Sponsor, SAEs in the following categories:

- Any death or hospitalisation due to new diagnosis or treatment of a cancer
- Any death or hospitalisation due to exacerbation of an existing medical condition
- SAEs due to expected side effects of biopsy as defined in the BAUS “Transrectal Ultrasound-Guided Biopsies of the Prostate Gland” leaflet

AEs will only be recorded in relation to study procedures, namely, MRI, biopsy procedure and post-biopsy events. Participants will either be called or approached at the time of their routine clinic visit approximately 1–2 weeks following the biopsy procedure to record biopsy-related AEs. It is anticipated that pain and bleeding will occur as a result of biopsy, but they will only be recorded as an AE if they continue for more than 4 days post biopsy.

### Patient and public involvement

The research question was discussed in a Tayside Urological Cancers Network (TUCAN) meeting with input from patients and public representation. The main attractions of the proposal for patients and the public were reducing the number of biopsies, improving detection of clinically significant cancers and avoiding having biopsies in men not needed. Patients helped in the preparation of a layman summary during the grant application. They contributed to the design of information and consent forms. We have advertised the study locally in meetings with the patients and public such as the Dundee Prostate Cancer Group and the Perth and Kinross prostate cancer and prostate diseases groups. The results will be summarised in a layman summary (briefing document) and widely publicised in local and national patients’ groups and prostate cancer charities. The advantages of the intervention were discussed in patients’ and public engagement meetings.

## Discussion

### Study conduct responsibilities

#### Protocol amendments, deviations and breaches

It is expected that the study protocol and other documentation may need further amendments during the lifetime of the proposed research. The CI will have a responsibility to inform and seek approval for any such proposed alterations to the protocol from the Sponsor, REC and NHS R&D Office(s). The changes thought to be necessary in the study protocol will not be executed without prior approval in place. The current protocol version is V10.0 issued on 24 January 2019.

It will be the responsibility of the CI to ensure documentation of any deviation from the protocol that may take place during the conduct of the trial. In case there is any deviation necessary, a clear reason for and the nature of this should be documented and communicated to the Sponsor. In case deviation merits an amendment in the study protocol, documentations need further submission to the Sponsor for approval and further appropriate action by the REC and NHS R&D Office to allow necessary reviews and approval.

Any serious breach of GCP during the study, if suspected, will be immediately reported to the Sponsor using the standard form “Notification to Sponsor of Serious Breach or Serious Deviation”.

#### Study record retention

Most of the source data within the study will form part of the patient’s clinical records. Data generated specifically for the study but of potential clinical relevance will be stored in the patient records and are thus subject to NHS Scotland minimum retention periods. In accordance with this, pathology and genetic data and samples will be kept for a minimum period of 30 years.

The research database which will bring together all relevant data, and which will not contain personal identifiers, will be kept for a period of 5 years after the end of the study. Records will then be deleted unless a review at the time suggests that a longer retention period is indicated. Access and security arrangements for the database will be the same as those during the study.

### Consenting participants

Timing of consent will differ depending on the local standard care pathway; however, at all sites, consent will be obtained prior to any study-related procedures commencing. Patient consent will be discussed with a suitably qualified member of the research team. Consent will only be taken if patients express a wish to participate. Consent will only be sought once a full discussion has taken place and the patient has expressed satisfaction with the information received. The person discussing the study with the patient will normally be the research nurse from the study team. A model consent form is shown in Additional file 2.

### Data access and confidentiality

The study team and the CI of the proposed trial will ensure compliance to the requirement of the Data Protection Act 1998. The core principles of the act such as the collection, processing of information, disclosure and long-term storage will be adhered to without exceptions. Given that the study involves a close collaboration with the NHS, the CI and study team will ensure adherence to the latest version of the NHS Scotland code of practice and procedures on patient confidentiality. Standard operating procedures such as limited access measures to computers via confidential log-in and password details will be followed to ensure restricted access to participant data by the CI. No personal data will be published through outcomes of the study, and the participants’ confidentiality will be ensured by not revealing any identification details of the individuals.

Participants’ identifiable details through imaging data, laboratory specimens, data entry forms, pathology reports and other records will be protected through institutional designed mechanisms. There will be access, on a principle of “limitation of purpose“ basis, to all records for study staff. Without prior written permission of the participants, no clinical details except when absolutely necessary for the purpose of study monitoring or auditing by the Sponsor will be disclosed or released by the CI or study team. The Sponsor or its representative will be informed in writing to obtain permission of any disclosure to third party, and without approval of the Sponsor no study confidential data, records of the participants or any other unpublished details will be disclosed.

### Ancillary and post-trial care

After the biopsy procedure, the clinical/research team will assess and record any experience of pain, haematuria and blood from the back passage. All criteria will be assessed during a telephone call or clinic visit approximately 7–14 days post biopsy. Patients will be given a diary sheet as an aide-mémoire to record any symptoms which will be used to complete the CRF via telephone call/clinic visit. In the event that a diary sheet is not completed/returned, the clinical/research team will review the clinical records to assess whether the participant has experienced any defined AEs and record this in the CRF. A model patient biopsy diary form is shown in Additional file 3.

### Dissemination

The results of this study will be used for publication and presentation in national and international journals and at scientific conferences.

### Roles and responsibilities

For roles and responsibilities of the trial sponsor, CI, PI and committees in this study, please see Additional file 4. This study protocol follows the 2013 Standard Protocol Items: Recommendations for Interventioanl Trials (SPIRIT) statement and the SPIRIT checklist is shown in Additional file 5.

## Trial status

The favourable approval for this trial was obtained from the East of Scotland REC 1 (Ref: 14/ES/1070) on 20 November 2014. The first patient was enrolled on 17 February 2015, with 416 participants so far recruited. The total duration of the recruitment phase was 48 months; however, this has now been extended to 66 months. The recruitment is scheduled to finish on 30 June 2020.

## Supplementary information

**Additional file 1:** Participant information sheet.

**Additional file 2:** MULTIPROS study, informed consent form.

**Additional file 3:** Patient’s biopsy diary.

**Additional file 4:** Roles and responsibilities.

**Additional file 5:** SPIRIT Checklist for this clinical trial.

## Data Availability

All datasets recorded and analysed in the MULTIPROS study are available on reasonable request from the corresponding author.

## Abbreviations

AE: Adverse event
CRF: Case report form
DRE: Digital rectal examination
HIC: Health Informatics Centre
mpMRI: Multiparametric magnetic resonance imaging
PCa: Prostate cancer
PIS: Patient information sheet
PSA: Prostate-specific antigen
R&D: Research and Development
REC: Research Ethics Committee
SAE: Serious adverse event
TRUS: Transrectal ultrasound
US: Ultrasound
