# Supplementary material for: A multicentre parallel-group randomised trial assessing multiparametric MRI characterisation and image-guided biopsy of prostate in men suspected of having prostate cancer: MULTIPROS study protocol
Source: Trials. 2019 Nov 21;20:638. doi: 10.1186/s13063-019-3746-0 (PMC6868804; doi:10.1186/s13063-019-3746-0)
Supplement: Supplementary file 1 — Additional file 1: Participant information sheet. [file 13063_2019_3746_MOESM1_ESM.pdf]

PARTICIPANT INFORMATION SHEET

The MULTIPROS Study - Multiparametric MRI characterisation and guided biopsy of the prostate in men suspected of having prostate cancer

We would like to invite you to participate in a research project which we believe is going to be of potential importance. However, before you decide whether or not you wish to participate, we need to be sure that you understand firstly why we are doing it, and secondly what it would involve if you agreed. We are therefore providing you with the following information. Please take time to read the following information carefully and be sure to ask any questions you have, and, if you want, discuss it with family and friends. We will do our best to explain and to provide any further information you may ask for now or later. You do not have to make an immediate decision.

Participation in the study will not alter your planned care and you will attend all your scheduled clinic appointments.

The results of the study will also be used as part of a MD (Doctor of Medicine) degree at the University of Dundee by our consultant radiologist/principal investigator of the MULTIPROS trial Dr Magdalena Szewczyk-Bieda.

WHAT IS THE PURPOSE OF THE STUDY?

There have been significant advances in imaging methods for prostate cancer over the past decades. New techniques have been introduced enabling more precise localisation of cancer within the prostate gland. Our study aims to assess the usefulness of two such techniques, namely a magnetic resonance imaging (MRI) called multiparametric (MP) MRI and ultrasound guided biopsy technique. We hope that the results of our study will help reduce the number of biopsies needed to diagnose prostate cancer and differentiate between cancers which may be aggressive and those which may not be as aggressive, as the latter can be simply monitored and may not be need radical treatment.

WHY HAVE YOU BEEN INVITED?

Your GP has referred you to the Urology Clinic for a biopsy, because they suspect you may be at risk of cancer of your prostate. The Urology team (doctor/nurse), who has examined you in the clinic and reviewed your GP letter, agrees that there is a risk that your problem may be due to prostate cancer and further tests are required to investigate this. One of the tests would be a biopsy of your prostate gland. The procedure is guided by an ultrasound probe inserted into your rectum (back passage) to allow images of your prostate to be displayed and viewed on screen, this will help the clinician obtain its size and volume.

MULTIPROS

NHS Tayside

Our research project aims to investigate whether performing MP-MRI before the biopsy and MRI image guided (FUSION) biopsy technique will help to diagnose prostate cancer and differentiate between aggressive and less aggressive cancer.

WHAT DOES THE STUDY ENTAIL?

If you decide to take part in this study, you will have an examination of your prostate gland performed before the biopsy. The examination is called MP-MRI and is done in the Radiology Department/Clinical Imaging Facility of the hospital. The biopsy will not be carried out on the same day as your MP-MRI.

If your MP-MRI examination did not show abnormality, you will continue to follow your routine prostate biopsy to find out whether you have cancer or not. This will be done using an ultrasound probe through your back passage. You will also be asked about your medication and if required, you will be given advice of any changes to your medicines prior to your biopsy. Please refer to the *Attending for a trans-rectal ultrasound (TRUS) +/- prostate biopsy* leaflet provided to you from the clinic

If your MP-MRI examination shows significant abnormality, using a computerised system, we will randomly (like flipping a coin) assign you to either the routine prostate biopsy (as above) or the routine prostate biopsy and MRI image guided (FUSION) biopsy. The FUSION biopsy will be done using a special equipment, which uses the MRI images to help the person taking biopsies find the cancer within your prostate gland. Normally your biopsy will be carried out under local anaesthetic. However, on occasion another type of anaesthesia may be more appropriate. If this is the case, your doctor will discuss this with you. The local standard practice will be followed and this may include a pre-assessment visit. You will be given the local NHS information leaflet with your NHS pre-assessment visit appointment letter. The results of the MP-MRI will be stored and used for clinical information

On the day of your biopsy appointment, the clinical/ research team will ask you questions about your biopsy experience. They will also give you a diary sheet to take home to help you record any symptoms you may experience after your biopsy. They will either call or meet you at a planned clinic appointment and record any discomfort you may have experienced at home after your biopsy.

If the biopsy results show that you have a cancer and you choose the option of surgery to remove the prostate gland (Prostatectomy), your whole prostate gland will be examined and we may compare the results with the images from the MP-MRI examination. The process may delay your Prostatectomy biopsy result by approximately 1 week but this will not affect your planned treatment.

To summarise, you will receive all the investigations and treatment you would normally have received and additionally you will have a MP-MRI examination prior to

biopsy (normally some patients will have MRI examination performed after the biopsy results are positive for cancers) and if allocated to the FUSION biopsy, you will have additional biopsies taken during your standard biopsy. The benefit for you will be that any additional information we get from the pre biopsy MP-MRI examination will be used to plan your treatment.

HOW MUCH OF MY TIME WOULD IT TAKE?

We will first contact you by the phone number you provided us with at the time of your first visit to the clinic or available to us through your records, to find out whether you would like to take part in this study. If you express interest in participating, during this telephone consultation we will ask you some questions about your general health and your past medical history to make sure you can safely undergo MRI examination.

If you decide to take part in this study, you will have one additional appointment prior to biopsies to undergo MP-MRI Study. This visit will replace clinical visit, which you were to make during your clinical care pathway for the suspected prostate cancer.

The pre-biopsy MP MRI will take approximately 1 hour and this will ensure that better information is obtained from your prostate without imperfections, which may occur due to a small amount of bleeding in your prostate, caused by the biopsy needle.

The TRUS biopsy will take approximately 20 minutes and if you are randomised to the TRUS and FUSION biopsy you may be at the clinic for another 5-10 minutes longer.

Please refer at the back of this document summarising your study journey.

EXPENSES AND PAYMENT

You will not receive any payment for taking part in this study. The travel expenses for the MRI visit will not be reimbursed as this visit replaces clinical visit which you were to make during your clinical care pathway for the suspected prostate cancer.

WHAT WILL I BE REQUIRED TO DO?

Before your MP-MRI, you will meet one of the research nurses who will consent you after full discussion has taken place and you agreed with the information received. You will then be seen by the radiographer, the person taking your scan, and she/he will help you to complete a checklist about pieces of metal or other objects that might stop you having the scan.

If at this visit you are found to have a history of a penetrative eye injury or exposure to metal fragments in your eye(s) you will be asked to consider having an eye x-ray prior to your MRI scan to establish safety. This can be performed at the main x-ray department at the same visit or prior to your MRI scan. You will be taken or

directed to the x-ray department. If the radiology staff establishes that you are unsafe to scan you will not have your MRI and you will not be able to participate in the study. With your consent, we will write to your GP informing them of your MRI safety status, as this information may be of benefit for your future health care needs. During the eye x-ray you will be exposed to in 8 days from natural sources in the environment. The risk from this level of exposure is minimal.

You will then be asked to change into hospital gown and trousers for the scan. You will be prepared for your scan, which will involve placing a cannula or small tube into your arm, to allow medication to be given into your vein; the first medication is to relax the muscles of your bowel, as its movement may impair the quality of images that will be produced. The second medication is a special contrast medium, called Gadolinium, which will help to show foci of cancer in your prostate gland on the images. You will then be asked to lie up on the scanning table and then will be moved into the centre of the scanner. During the scan, you will be able to speak to the radiographer. The scan will take pictures of your pelvis. As the scan is noisy you will be wearing hearing protection. After you have completed the scan you are free to go home.

You are encourage to contact the research team if you have any questions would like to answered prior to attending your MRI appointment or to inform the research team if you have change your mind about participating.

WHAT ARE THE POTENTIAL DISADVATAGES OF TAKING PART IN THE STUDY?

MRI scanning: This type of scan is very safe and does not use radiation. Some people, when being scanned, may feel a bit closed in but you will be in constant contact with the person performing the scan and you can come out at any time. The scanner is a bit noisy but you will be given ear protection which also plays music. The insertion of the cannula (needle) for the contrast medium can be briefly uncomfortable and can on occasion cause some bruising.

Contrast Medium: Gadolinium is the contrast agent used in the prostate MRI; it provides greater contrast between normal tissue and abnormal tissue in the prostate. Gadolinium looks clear like water and is non-radioactive. In a prostate MRI scan, a contrast agent injected into the bloodstream can provide information about blood supply to the prostate tissues. Usually, several scans are taken: one before the contrast agent is injected and at least one after. The pre-contrast and post-contrast images are compared and areas of difference are highlighted. Gadolinium has been used for years without any serious complications in thousands of patients. The FDA declared Gadolinium safe for use in MRI in 1988. A few side effects, such as mild headache, nausea and local burning, and on occasion a slight local skin rash can occur. Very rarely (less than one in a thousand), patients are allergic to Gadolinium. The Gadolinium used in MRI is many times safer than the iodine type contrast used in CT scans.

To administer Gadolinium, we need to ensure that your kidneys are working reasonably well. We will review your medical record to establish if you have had your renal function assessed in the in the past 6 months. If this not the case, we will obtain a 5ml blood sample when you attend the prostate assessment clinic to assess your kidney function

Muscle relaxant: The drug called Buscopan will also be given to you prior to the MRI scan to relax the muscles of your bowel as its movement may impair the quality of images. Buscopan will only be given to you after it is confirmed that it is safe for you to take it. The effects of the drug do not last long and will wear off before you leave the department.

You may experience mild side effects after the drug has been given such as dry mouth, nausea, photophobia, blurred vision or constipation.

Further information will be available in the MRI scan leaflet which will be sent with your MRI scan appointment letter.

If you are randomised to have additional samples taken during your biopsy, the biopsy will take longer but there will be no additional discomforts and side effects other than what you would normally experience during the biopsy.

WILL I BE GIVEN THE RESULTS OF THE SCAN?

Your scan will be examined by a radiologist. Prior to your scan you will be asked to give consent to be informed of any incidental finding found during your scan and agree that members of the research team can contact you and/or your GP and make any referral required for further investigation. The necessary steps would be taken for you to be treated according to current clinical practice. Your Urology doctor will discuss the results of the TRUS-guided biopsy with you.

WHAT ARE THE POTENTIAL ADVANTAGES OF TAKING PART IN THIS PROJECT?

By having an MRI scan, the potential is there to detect disease, which may have otherwise not have been detected and allows for early management. Also, MRI scan prior to biopsy will shorten the time between the biopsy and the start of the treatment if biopsies showed cancer cells in your prostate. There is no guaranteed benefit from taking part in the study but your participation contributes to medical science and possible future benefits.

WHAT WILL HAPPEN TO THE INFORMATION COLLECTED IN THE STUDY?

If you decide to participate in the study and sign the consent form, your GP will be informed about your participation.
There will be two sets of information obtained after you have had your MP-MRI scan. One set will be the MP-MRI scan images (and/or eye x-ray, if applicable) and the other, the research data obtained from those images.

Your anonymized research data including MRI images will be stored using a unique study code which is non-identifiable. All research data will be kept in a locked filing cabinet in a locked room. Any web-based data will be stored in a secure password protected central database at Health Informatics Centre, University of Dundee for a period of 5 years, after which the data will be destroyed. Only individuals directly involved with the study or maintenance of the database will have access to this data. Reports or publications of research findings will not contain information through which you can be identified.

The clinical MRI images (and/or eye x-ray, if applicable) obtained will be stored indefinitely using your name and unique hospital record number within the NHS clinical system and can be made available to specialist doctors for your future health care needs

We may be required to allow regulatory authorities, who ensure that research is being carried out in the correct manner, to inspect your records but they will not have access to your name or address.

Any information we obtain from you and your health records will remain strictly confidential. Information will be stored securely under conditions in keeping with the current data protection laws.

WHAT ARE MY RIGHTS?

Participation in this study is entirely voluntary and you are free to take part or to withdraw from the study at any time without having to give a reason or without this affecting your future medical care or your relationship with medical staff looking after you. If you decide to withdraw from the study, all identifiable data will be withdrawn but unidentifiable data will be retained. If you have any further questions please contact Dr Magdalena Szewczyk-Bieda or Professor Ghulam Nabi, their contacts details at the back of this Participant Information Sheet.

WHAT IF THERE IS A PROBLEM?

a. **Right to raise concerns.**

If you have any concerns about your participation in the study you have the right to raise your concern with a researcher involved in conducting the study or a doctor involved in your care.

b. **Right to make a complaint**

If you have a complaint about your participation in the study, you should first talk to a researcher involved in the study. However, you have the right to raise a formal complaint. You can make a complaint to a senior member of the research team or to the Complaints Officer for NHS Tayside.

Complaints and Feedback Team
NHS Tayside
Ninewells Hospital
Dundee DD1 9SY
Freephone: 0800 027 5507

Email: feedback.tayside@nhs.net

c. **Right to make a claim**

In the event that you think you have suffered harm as a result of your participation in the study there are no automatic financial compensation arrangements. However, you may have the right to make a claim for compensation against the University of Dundee or NHS Tayside. Where you wish to make a claim, you should consider seeking independent legal advice but you may have to pay for your legal costs.

d. **Insurance**

The University of Dundee and Tayside Health Board are Co-Sponsoring the study.

The University of Dundee maintains a policy of public liability insurance which provides legal liability cover in respect of damages, costs and expenses arising out of claims.

Tayside Health Board is a member of the NHS Scotland Clinical Negligence and Other Risks Insurance Scheme (CNORIS) which provides legal liability cover of NHS Tayside in relation to the study.

You should be aware that if you apply for health, life, travel or income protection insurance you may be asked questions about your health, including medical history, pre-existing medical conditions, if you have had a genetic test or about your

participation in this study. It is not anticipated that your involvement in the study will adversely affect your ability to purchase insurance but some insurers may use this information to limit the offer of cover, apply exclusions or increase any premium. If you have a diagnosed medical condition, even where the condition is diagnosed as part of a research study, the insurer may take this in to consideration when deciding whether to offer insurance to you.

WHO HAS REVIEWED THIS STUDY?

The East of Scotland Research Ethics Committee 1, which has responsibility for scrutinising proposals for medical research on humans, has examined the proposal and has raised no objections from the point of view of medical ethics.

It is a requirement that your records in this research, together with any relevant medical records, be made available for scrutiny by monitors from University of Dundee and NHS Tayside, whose role is to check that research is properly conducted and the interests of those taking part are adequately protected.

WHAT HAPPENS NEXT?

If you are interested in taking part in the study, please complete the information in the contact section at the end of this information sheet; this is not consent to participate in the study but just your intention to consider it. A research nurse will contact you in the next few days on the number you have provided to discuss the study with you. During this telephone conversation there will be opportunity to ask all the questions you may have. If you state your intention to participate in the study, you will be invited for a consent interview, during which you will again have opportunity to have all your questions answered before you sign a consent form to participate. You will also be asked some questions to ensure you are eligible for MRI.

Alternatively, please call a member of the research team (details below) to discuss the study and arrange an appointment or let your Urology doctor know you would like to be contacted about the study.

WHO IS ORGANISING AND FUNDING THIS RESEARCH?

The study has been reviewed and funded by Prostate Cancer UK, a UK based charity and Chief Scientist Office, Scotland through a competitive bid. The University of Dundee and Tayside Clinical Trial Unit are organising this study.

Thank you for reading this information sheet and considering taking part in this study

For further information, please visit the MULTIPROS website at multipros.org.uk

STUDY TEAM CONTACT DETAILS

Clinical Research Nurse
Debbie Forbes
Stefani Unit, Ward 32
Ninewells Hospital & Medical School
Dundee, DD1 9SY
Tel: 01382 496685
Email: debbieforbes@nhs.net or crcstudies.tayside@nhs.net

Principal Investigator:
Dr Magdalena Szewczyk-Bieda
Consultant Radiologist and MD Student at
University of Dundee
Ninewells Hospital & Medical School
Dundee, DD1 9SY
Email: m.szewczyk-bieda@nhs.net

Chief Investigator:
Professor Ghulam Nabi
Professor in Surgical Uro-Oncology
Honorary Consultant Urological Surgeon
Head of Division of Imaging Sciences and Technology
Lead for Minimally Invasive Surgery in Urological Cancers
Chair Tayside Urological Cancer Network (TUCAN)
Ninewells Hospital & Medical School
Dundee, DD1 9SY
Email: g.nabi@dundee.ac.uk

If you become unwell during the study and need urgent advice or assistance please contact the usual NHS services such as NHS24 (Tel: 111) or contact your GP.

Summary of Participant Care Journey

VISIT

- Attend prostate clinic for prostate assessment
- Receive Study Participant Information Sheet

↓

TELEPHONE CALL

- Telephone call from Research Nurse to confirm if you wish to take part in the study
- Check Safety Criteria for MRI Scan
- If interested, Research Nurse will arrange MRI appointment

↓

VISIT

- Attend MRI scan
- Complete Study Consent Form

↓

VISIT

- Attend Prostate Biopsy
- If MRI is negative, you will receive the routine prostate biopsy
- If MRI positive, Research Team will randomise you to receive either the routine prostate biopsy or the routine prostate AND FUSION biopsies
- Take home participant diary to record any symptoms occurring during and after your biopsy

↓

VISIT

- Return for clinic visit to receive the results of your biopsy
- Return your participant diary

CONTACT SECTION:

Please fill the attached intention to consider participation in this study section if you would like to consider participation in this study.

If you are certain you don't wish to participate, simply hand the leaflet back to reception. You don't need to fill anything.

The Identifiable information you provided will be held on a password-protected database in the University of Dundee in accordance with the Data Protection Act 1998.

Intention to consider participation in this study.

I would like to consider participation in MULTIPROS study and I am happy to be contacted on the number provided below by a member of the research team.

NAME:……………………………………………………………

DATE OF BIRTH: ……………………………………….……...

TELEPHONE NUMBER:………………………………………..

PREFERRED TIME OF A DAY TO BE CONTACTED:

……………………………………………………………………………
