# Supplementary material for: A multicentre parallel-group randomised trial assessing multiparametric MRI characterisation and image-guided biopsy of prostate in men suspected of having prostate cancer: MULTIPROS study protocol
Source: Trials. 2019 Nov 21;20:638. doi: 10.1186/s13063-019-3746-0 (PMC6868804; doi:10.1186/s13063-019-3746-0)
Supplement: Supplementary file 2 — Additional file 2: MULTIPROS study, informed consent form. [file 13063_2019_3746_MOESM2_ESM.pdf]

MULTIPROS study

INFORMED CONSENT FORM

**Subject ID:**							

Subject ID- please use T for Tayside cohort and consecutive numbers 0001- 0600 followed by participant's initials

Title of Project: Multiparametric MRI characterisation and guided biopsy of the prostate in men suspected of having prostate cancer

Name of the Chief Investigator: Professor Ghulam Nabi

			INITIALS
1. I confirm that I have read and understood the Patient Information Sheet, V8.0 240119 for the above study. I have had the opportunity to consider the information, ask questions and have these answered satisfactorily.	YES ☐	NO ☐	
2. I agree to have MRI with contrast.	YES ☐	NO ☐	
3. I agree, if required, to have an x-ray of my eyes prior to MRI scan for MRI safety reasons.	YES ☐	NO ☐	
4. I understand that by undergoing an eye x-ray, the results may exclude me from having an MRI for safety reasons.	YES ☐	NO ☐	
5. I agree, if required, to be assessed for general and/or epidural anaesthesia.	YES ☐	NO ☐	
6. I understand that after my assessment, general or epidural anaesthesia may not be suitable for me and that I may be excluded from taking part in the study.	YES ☐	NO ☐	
7. I agree that my GP be informed of MRI safety status and of my participation in this study.	YES ☐	NO ☐	
8. I understand that my participation is voluntary and that I am free to withdraw at any time without giving any reason, without any medical care or legal rights being affected.	YES ☐	NO ☐	
9. I understand that my identifiable MRI images of my prostate gland will be stored within the NHS clinical system and will be available to specialist doctors looking after me in the future.	YES ☐	NO ☐	

INITIALS

10. I agree to be informed of any incidental finding found during my MRI scan and agree that members of the research team can contact both me and my GP and inform any referral specialist required to carry out further investigations.	YES ☐ NO ☐	
11. I understand that I may have additional samples taken during my biopsy procedure, and that the additional biopsies will be guided by MRI images from my MRI study.	YES ☐ NO ☐	
12. I understand that if my biopsy result shows I have cancer and I choose the option to have surgery to remove my prostate gland (prostatectomy) my prostate gland may be examined by the research team and the results will be compared with the MRI images.	YES ☐ NO ☐	
13. I understand that if I want to withdraw from the study, all identifiable data, not including my MRI scans, or tissue collected, would be withdrawn from the study. Data or tissue which is not identifiable to the research team may be retained.	YES ☐ NO ☐	
14. I understand that relevant sections of my medical notes and data collected during the study may be looked at by research team, sponsors or regulatory authorities where it is relevant to my taking part in this research. I give permission for the research team, sponsors or regulatory authorities to have access to my records and data.	YES ☐ NO ☐	
15. I understand that personal data about me and research data collected during the study will be stored by the University of Dundee and that I have the right to request that the personal and/or research data is deleted.	YES ☐ NO ☐	
16. I understand that my research data collected by the research team in this study may be used to support other research in the future, and may be shared anonymously with other researchers or collaborators.	YES ☐ NO ☐	
17. I agree to take part in the above study.	YES ☐ NO ☐	

Name of Participant	Date	Signature

Name of Person taking consent	Date	Signature

1 copy for participant; 1 copy for medical notes; 1 (original) to be kept in Site Investigator File
