# Supplementary material for: A multicentre parallel-group randomised trial assessing multiparametric MRI characterisation and image-guided biopsy of prostate in men suspected of having prostate cancer: MULTIPROS study protocol
Source: Trials. 2019 Nov 21;20:638. doi: 10.1186/s13063-019-3746-0 (PMC6868804; doi:10.1186/s13063-019-3746-0)
Supplement: Supplementary file 3 — Additional file 3: Patient’s biopsy diary. [file 13063_2019_3746_MOESM3_ESM.pdf]

MULTIPROS

NHS Tayside

Patient's Biopsy Diary

G/A/SA ☐	Subject ID/Study number :	T/G	0					

Please complete diary as discussed with the research team.

This information will discussed during your follow-up call or clinic visit.

Please refer to the Prostate Scan Leaflet given to you at your biopsy appointment if you require further information or the contact for advice.

Please use these descriptions to help you complete the **Severity** section in the form.

Mild - mild discomfort, no limitation to daily activities, no medical/therapy intervention required (e.g. no pain relief)

Moderate - Mild to moderate limitation in activity, medical/therapy intervention required (e.g. required pain relief)

Severe - Marked limitation in activity, medical/therapy intervention required (e.g. required pain relief, seen by GP)

Examples of **Other** symptoms include urine infection. If you have more symptoms please add them to the back of this form.

**Have you experienced any of the following during/after biopsy?**					
	**Pain** **within 24 hours of biopsy**	**Pain** **after 24 hours of biopsy**	**Blood in Urine after biopsy**	**Blood from back passage after biopsy**	**Other, specify**
	Yes ☐ No ☐	Yes ☐ No ☐	Yes ☐ No ☐	Yes ☐ No ☐	
**Severity**	☐ Mild<br>☐ Moderate<br>☐ Severe	☐ Mild<br>☐ Moderate<br>☐ Severe	☐ Mild<br>☐ Moderate<br>☐ Severe	☐ Mild<br>☐ Moderate<br>☐ Severe	☐ Mild<br>☐ Moderate<br>☐ Severe
**Duration**	☐ Only during Biopsy<br>☐ Up to 1 hour after biopsy<br>☐ Up to 5 hours<br>☐ More than 5 hrs but less than 24 hours	☐ More than 24 but less than 48hrs<br>☐ 2-3 days<br>☐ 4-7 days<br>☐ More than 7 days	☐ Less than 24 hrs<br>☐ 1- 3 days<br>☐ 4-7 days<br>☐ More than 7 days	☐ Less than 24 hrs<br>☐ 1-3 days<br>☐ 4-7 days y<br>☐ More than 7 days	☐ Less than 24 hrs<br>☐ 1-3 days<br>☐ 4-7 days<br>☐ More than 7 days
