# Supplementary material for: A multicentre parallel-group randomised trial assessing multiparametric MRI characterisation and image-guided biopsy of prostate in men suspected of having prostate cancer: MULTIPROS study protocol
Source: Trials. 2019 Nov 21;20:638. doi: 10.1186/s13063-019-3746-0 (PMC6868804; doi:10.1186/s13063-019-3746-0)
Supplement: Supplementary file 4 — Additional file 4: Roles and responsibilities. [file 13063_2019_3746_MOESM4_ESM.docx]

**Roles and Responsibilities**

**Trial Sponsor**: NHS Tayside/University of Dundee

Sponsor’s reference: 2013ON23

Contact Name: Tricia Burns, Senior Research Governance Manager

Address: Tayside Medical Science Centre, (TASC), Ninewells Hospital & Medical School Residency Block Level 3, George Pirie Way, Dundee, DD1 9SY

Telephone: 01382 383890

Email: [p.burns@dundee.ac.uk](mailto:p.burns@dundee.ac.uk), [tascgovernance@dundee.ac.uk](mailto:tascgovernance@dundee.ac.uk)

The trial is funded by Prostate Cancer UK. NHS Tayside/University of Dundee has taken the sponsorship responsibilities and will meet the standards as laid down in the Research Governance Framework.

Role: The sponsor initially and will continually assess the trial meets and conforms with the law and in accordance with the best current practices. Any changes to the trial protocol or supporting documents will need to be submitted and approved by the sponsor. Submission to the appropriate regulatory organization will take place after the sponsor has given approval.

**Chief Investigator: Professor Ghulam Nabi**

Address: Ninewells Hospital and Medical School Dundee, DD1 9SY

Telephone: 01382 383192

Email: [g.nabi@dundee.ac.uk](mailto:g.nabi@dundee.ac.uk)

Role: Responsible for the conduct of the whole project. Ensure the funding is in place for the duration of the trial. Advised PI and assist with trial queries. Make certain the protocol, standard operating procedures and working practices are in place and adhered to. Ensure the information technology used for the trial is adequately designed, appropriately located, safe and suitable for the purpose. Co-operate with all regulatory authorities in the conduct of audits and inspections.

**Principal Investigator:**

Contact Name:

Ninewells Hospital - Dr Magdalena Szewczyk-Bieda, [m.szewczyk-bieda@nhs.net](mailto:m.szewczyk-bieda@nhs.net)

Aberdeen Royal Infirmary – Dr Senthil Ragupathy, [sarcotragupathy@nhs.net](mailto:sarcotragupathy@nhs.net)

Royal Free Hospital – Mr Paras Singh, [paras.singh@nhs.net](mailto:paras.singh@nhs.net)

Role: The Principal Investigator (PI) at each participating site is responsible for the overall conduct of the trial. It includes the safety and welfare of the participants and the consistency of the data generated from the site. The PI is principally responsible for the identification, recruitment, data collection and completion of the paper Case Report Form (pCRF) and electronic Case Report Form (eCRF). The PI is responsible to ensure the site personnel involved in the trial has the relevant skills, expertise and time to complete the tasks. The PI is also responsible to ensure the site are able to provide the necessary facilities and equipment required to conduct the trial procedures and investigations.

**Trial Management Group (TMG):** The TMG is made up of the Chief Investigator, Trial Manager, Trial Coordinator, Principal Investigators, Research Nurses, Trial Co-Investigator, Statisticians, and Senior Trial Manager.

Role: Trial planning. Preparation of trial documents and revisions. Submission of documents for governance and regulatory approval. Organization of the Joint Trial Steering Committee and Data Management Committee. Prepare and submit required reports. Monitor participant’s recruitment, safety, and breaches. Discuss solutions to the trial challenges. Data management. Analysis and interpretation of data. Maintenance of the trial systems. Trial publication.

**Joint Trial Steering Committee and Data Management Committee (Joint TSC and DMC):**

The joint committee comprises of an independent chair and a minimum of two other independent expert members. The CI, co-investigators and members of the TMG or other relevant experts are invited to attend as appropriate.

Role: The joint committee oversees the progress of the study. The group reviews the trial targets, outcomes, and milestones. The group informs the TMG of external factors that may have an impact on the success of the trial and encourages the TMG group to develop strategies to deal with recruitment problems. Also comments on significant changes to the protocol, manuscript, publication.
