# Supplementary material for: A multicentre parallel-group randomised trial assessing multiparametric MRI characterisation and image-guided biopsy of prostate in men suspected of having prostate cancer: MULTIPROS study protocol
Source: Trials. 2019 Nov 21;20:638. doi: 10.1186/s13063-019-3746-0 (PMC6868804; doi:10.1186/s13063-019-3746-0)
Supplement: Supplementary file 5 — Additional file 5: SPIRIT Checklist for this clinical trial. [file 13063_2019_3746_MOESM5_ESM.docx]

| SPIRIT CHECKLIST | | | page |
| --- | --- | --- | --- |
| [1-5] ADMINISTRATIVE INFORMATION | 1: TITLE | | 1 |
|  | 2: TRAIL REGISTRATION | 2A: REGISTRY | 2 |
|  |  | 2B: DATA SET | 2* |
|  | 3: PROTOCOL VERSION | | 10 |
|  | 4: FUNDING | | 13 |
|  | 5: ROLES AND RESPONSIBILITIES | | 11 |
| [6-8] INTRODUCTION | 6: BACKGROUND AND RATIONALE | | 3 |
|  | 7: OBJECTIVES | | 3 |
|  | 8: TRIAL DESIGN | | 3 |
| [9-15] METHODS: PARTICIPANTS, INTERVENTIONS, OUTCOMES | 9: STUDY SETTING | | 4 |
|  | 10: ELIGIBILITY CRITERIA | | 5-6 |
|  | 11: INTERVENTIONS | | 6 |
|  | 12: OUTCOMES | | 7 |
|  | 13: PARTICIPANT TIMELINE | | 3, figure1 |
|  | 14: SAMPLE SIZE | | 4 |
|  | 15: RECRUITMENT | | 4 |
| [16-17] METHODS: ASSIGNMENT OF INTERVENTIONS (FOR CONTROLLED TRIALS) | 16: ALLOCATION | | 6 |
|  | 17: BLINDING (MASKING) | | 6 |
| [18-20] METHODS: DATA COLLECTION, MANAGEMENT, ANALYSIS | 18: DATA COLLECTION METHODS | | 7-8 |
|  | 19: DATA MANAGEMENT | | 7-8 |
|  | 20: STATISTICAL METHODS | | 7 |
| [21-23] METHODS: MONITORING | 21: DATA MONITORING | | 8-9 |
|  | 22: HARMS | | 8-9 |
|  | 23: AUDITING | | 8 |
| [24-31] ETHICS AND DISSEMINATION | 24: RESEARCH ETHICS APPROVAL | | 2, 13 |
|  | 25: PROTOCOL AMENDMENTS | | 10 |
|  | 26: CONSENT OR ASSENT | | 10 |
|  | 27: CONFIDENTIALITY | | 10-11 |
|  | 28: DECLARATION OF INTERESTS | | 13 |
|  | 29: ACCESS TO DATA | | 13 |
|  | 30: ANCILLARY AND POST-TRIAL CARE | | 11 |
|  | 31: DISSEMINATION POLICY | | 11 |
| [32-33] APPENDICES | 32: INFORMED CONSENT MATERIALS | | 10 |
|  | 33: BIOLOGICAL SPECIMENS | | N/A |

*: Please refer to Item 2A and registration information in <https://clinicaltrials.gov/ct2/show/NCT02745496>
